# Supplementary material for: Dynamic 3D imaging of cerebral blood flow in awake mice using self-supervised-learning-enhanced optical coherence Doppler tomography
Source: Commun Biol. 2023 Mar 21;6:298. doi: 10.1038/s42003-023-04656-x (PMC10030663; doi:10.1038/s42003-023-04656-x)
Supplement: Supplementary file 2 — Supplementary Information [file 42003_2023_4656_MOESM2_ESM.pdf]

Supplementary Information

Supplementary S1: Sensitivities of µOCA, µODT to motion noise

Supplementary Note S1: Since the sensitivity of µODT is lower than that of µOCA (e.g., more sensitive to background phase noise, stage vibration) based on fundings from previous anesthetized animal imaging studies, we expected that the deterioration of µODT images in awake animals would be more serious. However, results showed that µOCA was more severely affected by bulk motion artifacts in the awake animal. This phenomenon can be explained by the formulas used to reconstruct µOCA and µODT images. Take phase subtraction method (PSM) for simplicity, the flow rate at *v(x_i, Δz)* of µODT can be given as

$$v_i = \frac{\Delta\varphi_i \lambda_0}{4\pi n\tau \cos\theta_z} \qquad (s1)$$

where k is wave number, λ_0 is central wavelength of the light source, n≈1.38 is refractive index of brain tissue, Δz is depth from brain surface, θ_z is incline angle of flow, and $\Delta\varphi_i$ is the phase difference

$$\Delta\varphi_i = \varphi[F^{-1}[I_{i+1}(k,\Delta z)] - \varphi[F^{-1}[I_i(k,\Delta z)] \qquad (s2)$$

between 2 adjacent A scans at I_{i+1} or $I(x_{i+1}, \Delta z)$ and I_i or $I(x_i, \Delta z)$ of τ=Δt time interval.
The vasculature of µOCA can be given as

$$A(x,z) = \frac{1}{\bar{I}(k,\Delta z)} \frac{1}{N-1} \left[\sum_{j=1}^{N} \left[I_j(k,\Delta z) - \bar{I}(k,\Delta z) \right]^2 \right]^{\frac{1}{2}} \qquad (s3)$$

where N is the B-scan numbers (e.g., N=4), $\bar{I}(k,\Delta z)$ is the mean intensity of the cross-section, i.e.,

$$\bar{I}(k,\Delta z) = \frac{1}{N} \sum_{i=1}^{N} I_i(k,\Delta z) \qquad (s4)$$

the normalized standard deviation among N B-scans. If we assume the speed of bulk motion artifacts is v_{BMA}, the resultant displacement $\Delta S_{ODT} \approx v_{BMA} \times \Delta t_A$ is significantly less than that of $\Delta S_{OCA} \approx v_{BMA} \times \Delta t_B$, where Δt_A, Δt_B are the duration between 2 adjacent A-scan in µODT and B-scan in µOCA, respectively. For example, $\Delta t_A \approx 1/6 \times 10^{-3}$s, $\Delta t_B \approx 1/27$s; if considering 14 A-scan oversampling for µODT in Eq.(r1) and N≈6 B-scans in Eq.(r4) for µOCA, $\Delta t_A \approx 14/6 \times 10^{-3}$s, $\Delta t_B \approx 6/27$s. In other words, the resultant of motion artifacts on µOCA is roughly $\Delta t_B/\Delta t_A \approx 200$x higher than on µODT. Therefore, the bulk motion artifacts of awake animal are more serious in µOCA than in µODT images as we observed and the results of machine learning denoising are more obvious.

Supplementary S2: Experiments and animal groups used in this study

Supplementary Table S1(a) Experiments and animal groups to compare flows in anesthetized vs awake states

Experiment Descriptions	Total Experiments (Animal #)	Drug Challenge
Awake to Isoflurane anesthesia transition	11 (7)	Inhalational isoflurane (2% in O_2)
Awake to Dex anesthesia transition	11 (3)	Dexmedetominine hydrochloride (0.025mg/kg, i.p.)
Awake to ketamine & xylazine anesthesia transition	4 (3)	Ketamine & Xylazine (87.5mg/kg ketamine & 12.5mg/kg xylazine, i.p.)

Supplementary Table S1(b) Experiments and animal groups to study CBFv response to cocaine in anesthetized vs awake states

Experiment Descriptions	Total Experiments (Animal #)	Drug Challenge
Cocaine effect in awake animals	10(7)	Cocaine injection (1mg/kg, i.v.)
Cocaine effect in Isoflurane anesthetized animal	7(4)	
Cocaine effect in Dex anesthetized animals	6(2)	
Cocaine effect in ketamine/xylazine anesthetized animals	4(3)	

Supplementary S3: Experimental procedures with ketamine as anesthetic and results

The details of experimental procedures are summarized as follows:

1). Full-field awake μOCA/μODT scan: FOV=2.25x2.0x1.2mm^3;

2). Dynamic μODT scans in the transition from awake to ketamine states: 2.25x0.3x1.2mm^3/volume per 1.5-2min over 40min, including ~10min baseline to 10min after *i.p.* injections of anesthetic cocktails, e.g., an initial dose of ketamine (87.5mg/kg) + xylazine (12.5mg/kg), following-up doses of ketamine (29.2mg/kg) + xylazine (4.2mg/kg);

3). Full-field ketamine anesthetized μOCA/μODT scan: FOV=2.25x2.0x1.2mm^3;

4). Dynamic μODT scans of cocaine effects: 2.25x0.3x1.2mm^3/volume per 1.5-2min over 40min, including ~9-10min baseline to 36min after cocaine injection (1mg/kg, i.v.) during which follow-up i.p. doses of ketamine (29.2mg/kg) + xylazine (4.2mg/kg) were infused;

5). Full-field ketamine anesthetized μOCA/μODT scan after cocaine: FOV=2.25x2.0x1.2mm^3.

Due to the short lifetime (~20-30min) of ketamine/xylazine cocktail for anesthetics, additional 3 injections were given for each experiment after the initial dose to stabilize the animal for experiments.

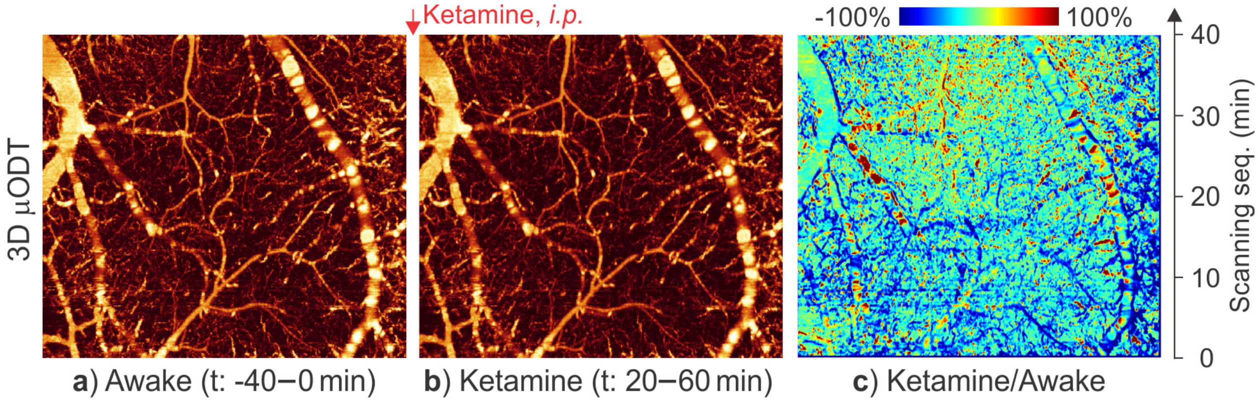

Supplementary Fig.s1 Comparison of 3D μODT images of CBFv networks in mouse sensorimotor cortex in awake (**a**) vs ketamine-anesthetized (**b**) states, and their ratio image (**c**), showing unstable CBFv changes under ketamine anesthesia (c). Image size: 2.25×2×1.2mm^3.

Supplementary Fig.s1 shows a pair of representative 3D μODT images of the CBFv networks of mouse cortex in awake (**a**) vs ketamine-anesthetized (**b**) states. Their ratio image (**c**) reveals that ketamine-induced CBFv decreased in the first 20min post ketamine injection (blue regions) followed by a partial recovery with a slight overshooting (yellow and red regions) as a result of short lifetime of ketamine anesthesia. Despite the complications, ketamine anesthesia in general resulted in regional CBFv decreases.

To further track cocaine-induced CBFv dynamic changes in the cortex of ketamine-anesthetized animals, similarly a smaller panel of 2.25×0.3×1.2mm^3 highlighted by a dashed yellow box in **Fig.s2(a)** was selected to acquire time-lapse 3D μODT images in **Fig.s2(d)** and their ratio images in **Fig.s2(e)**. **Fig.s2(f)** plots the relative flow changes in individual vessels (dashed traces, m=16), showing cocaine-induced inhomogeneous CBFv responses, e.g., increasing and decreasing fluctuations in arteriolar and venular flows, especially in capillaries (green traces). Such temporally inhomogeneous flow responses to cocaine are consistent with their spatially inhomogeneous responses shown in **Fig.s2(c)**. **Fig.s2(g)** summarizes the comparison of CBFv changes in ketamine anesthetized state before and at 28min after cocaine across animals (ROIs=14-16/animal, n=3), showing no significant difference (p=0.57). The inhomogeneous responses to cocaine in the neurovascular network might imply the confounding effects of ketamine on neuronal activities and cerebral hemodynamics.

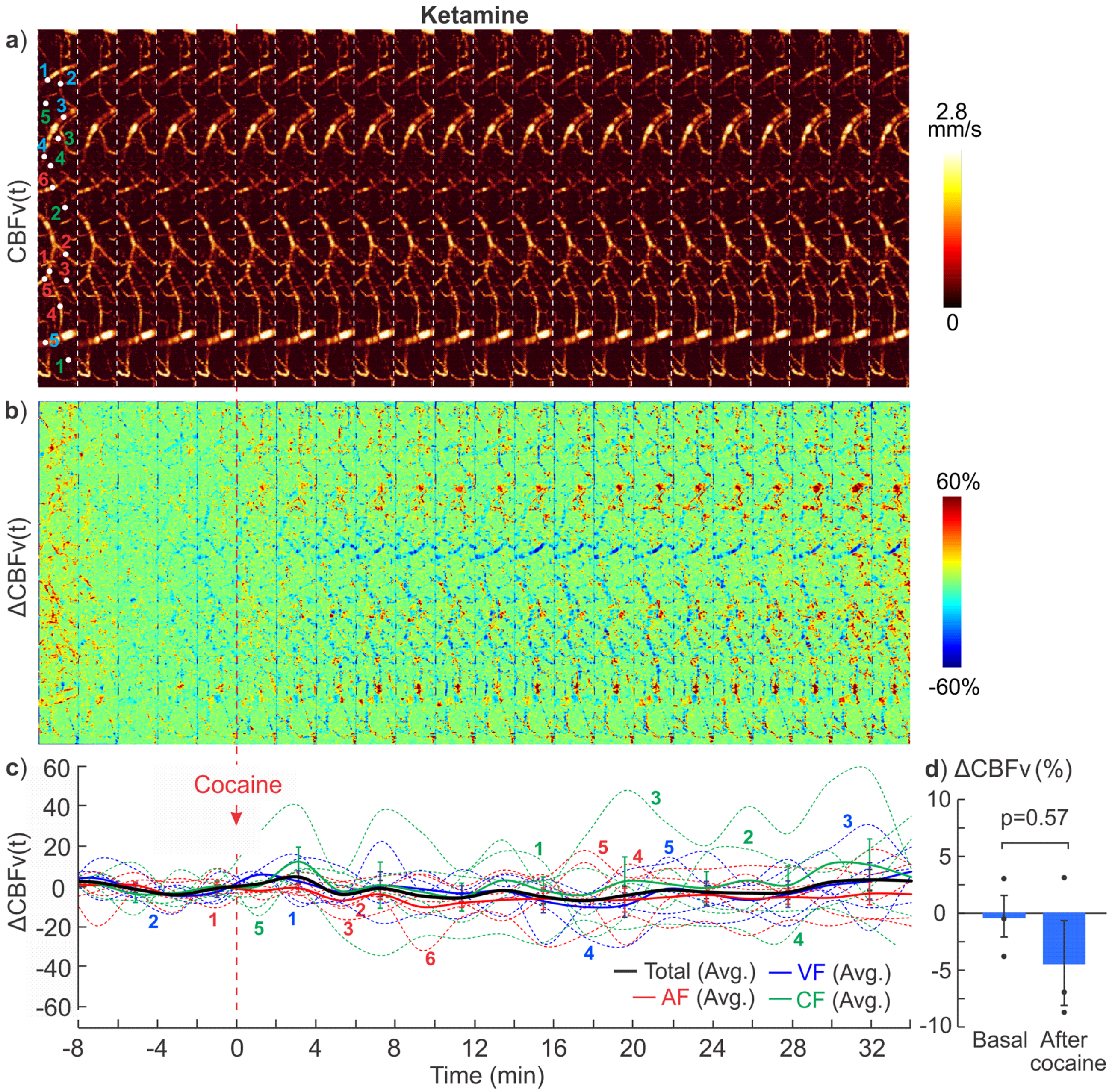

Supplementary Fig.s2 CBFv responses to cocaine in ketamine-anesthetized animal. **a-b**): time-lapse images μODT(t) and before and after cocaine (1mg/kg, i.v. at t=0min) and their ratio changes ΔμODT, image size: 2.25×0.3×1.2mm^3; **c**) cocaine-induced CBFv changes of individual vessels; **d**) Statistical comparison of CBFv changes before and after cocaine (ROIs=14-16/animal, n=3), showing no significant change (p=0.57).

Supplementary S4: Small regions selected for time-lapse imaging of CBFv dynamic changes in response to cocaine

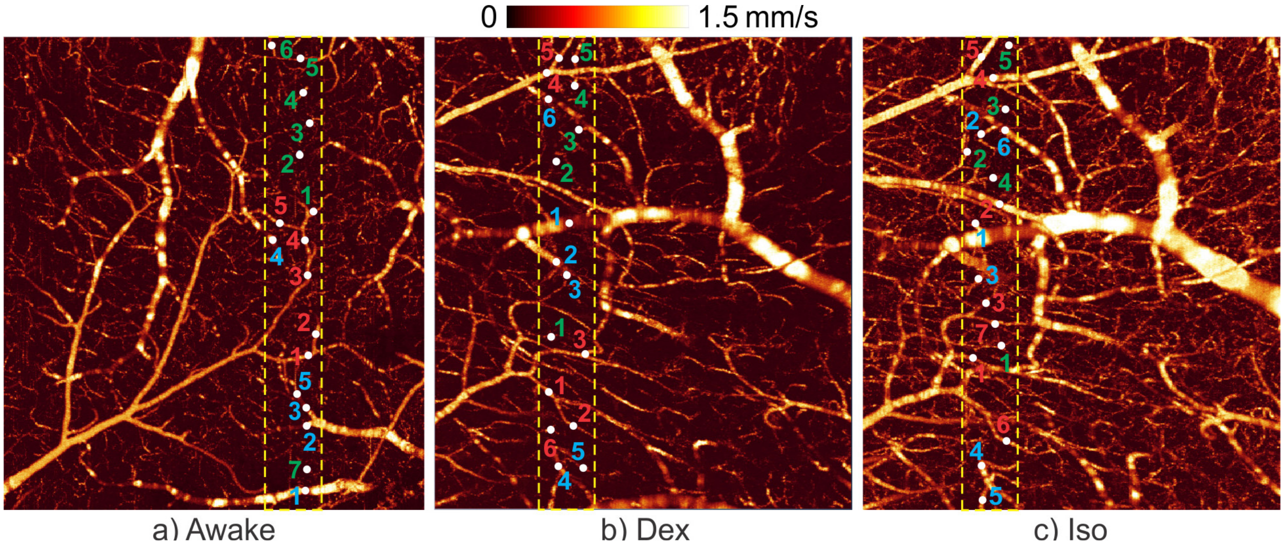

Supplementary Fig.s3 Full-field 3D μODT images of mouse cortex in awake (a) vs Dex (b) and isoflurane (c) anesthetized states (image size: $2.3\times2\times1.2mm^3$). Dashed yellow boxes landmarked the ROIs ($2.25\times0.3\times1.2mm^3$) for fast time-lapse 3D μODT imaging of cocaine-induced CBFv(t) in **Fig.7**(**a**, **d, h**) and the solid red, light blue, and green dots of RIOs for sampling arteriolar, venular, and capillary CBFv changes.

Supplementary S5: Chronic cocaine elicited vasoconstriction and capillary flow density decreases

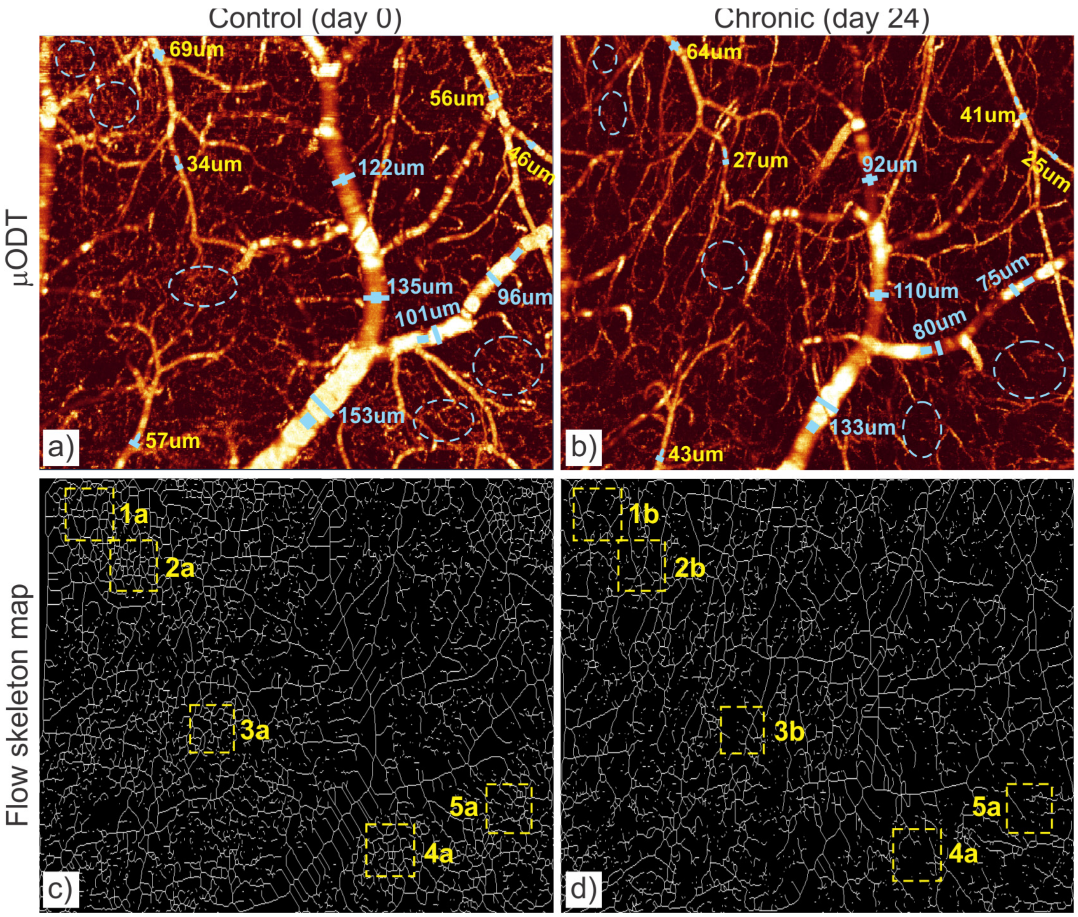

Supplementary Fig.s4 (**a**, **b**): 3D μODT images (2.3×2×1.2mm^3) of awake mouse cortex on day 0 (control) and day 24 (chronic cocaine). (**c**, **d**): the corresponding microcirculatory flow skeleton maps based on flow segmentation, binarized map generation and skeletonization[29,45]. The dashed yellow ROIs (1, …, 5) were used to calculate capillary flow densities in the corresponding dashed blue ROIs in (**a**, **b**).

Supplementary S6: Self-supervised deep learning to denoise motion artifacts of awake animal and to track animal movement (e.g., motion severity index)

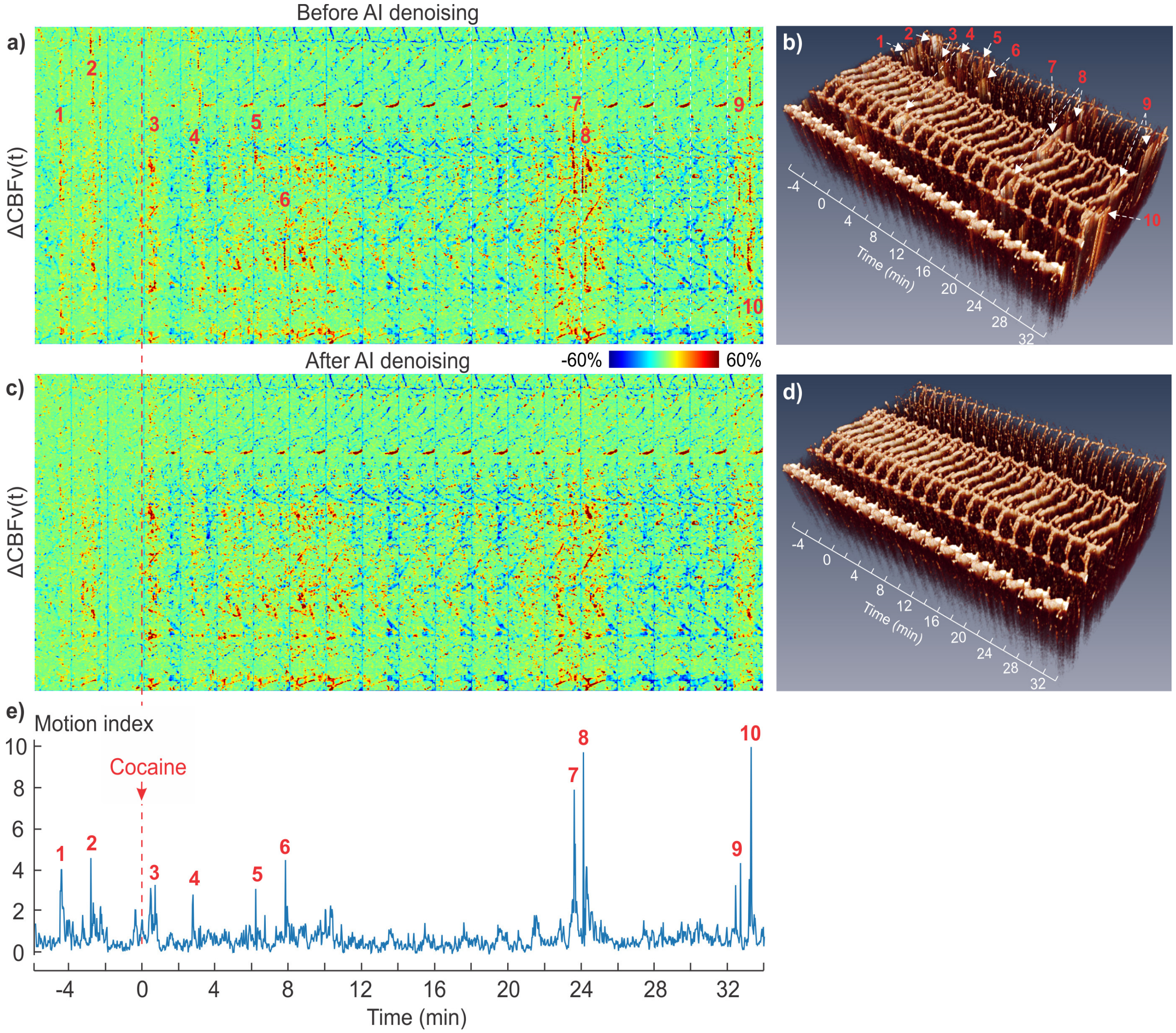

Supplementary Fig.s5 Effectiveness of self-supervised AI denoising to minimize motion artifacts (**c-d** vs. **a-b**) and to monitor animal movement (**e**: motion index). **a-b**): time-lapse images ratio changes ΔμODT (%) of raw **Fig.7b** and their 3D μODT(t) images to illustrate motion artifacts occurring at cross sections of i=1 to 10 without AI denoising, image size: 2.25×0.3×1.2mm^3; **c-d**): the corresponding images of (**a-b**) after AI denoising to effectively remove motion artifacts of animal movements – presented in **Fig.7b**. **e**): Motion index (i.e., decorrelation) to illustrate the timepoints or B-scans (i=1, …,10) of animal movement severity. This parameter can be used to monitor the associated motor function of awake animal.

Supplementary S7: Self-supervised vs prior supervised deep learning to denoise 3D μODT images in awake animal

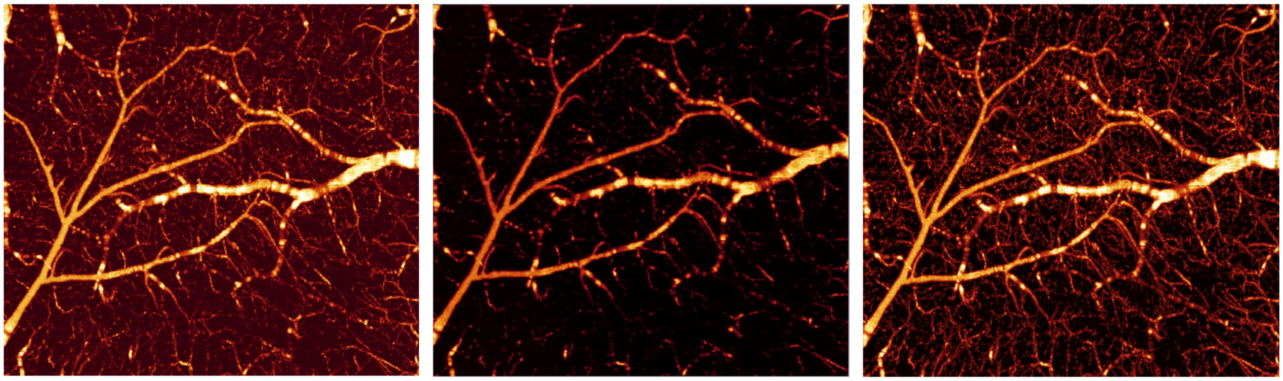

a) Raw image **b**) Supervised denoising (Li, et al) **c**) Self-supervised denoising

Supplementary Fig.s6 Comparisons of MIP images of raw μODT (**a**), denoised μODT using prior supervised deep learning[40], and using self-supervised deep learning in this study (**c**). The prior supervised denoising method produces high-contrast image of large, high flows but misses most capillary flows (**b**), whereas the current self-supervised denoising effectively reduces flow noises and retrieves high-contrast weak capillary flows whose flow patterns and flow rates are well preserved compared to the original image (**a**). This is likely due to the fact that prior supervised learning for denoising and bulk motion artifact removal of an awake animal replied on using images ('ground truth') of anesthetized animals acquired from the previous μODT setup to train the framework. Meanwhile, the self-supervised approach was more efficient and less system and animal physiology dependent.

Supplementary S8: CBFv network redistribution in the cortex of an awake mouse after chronic cocaine exposure

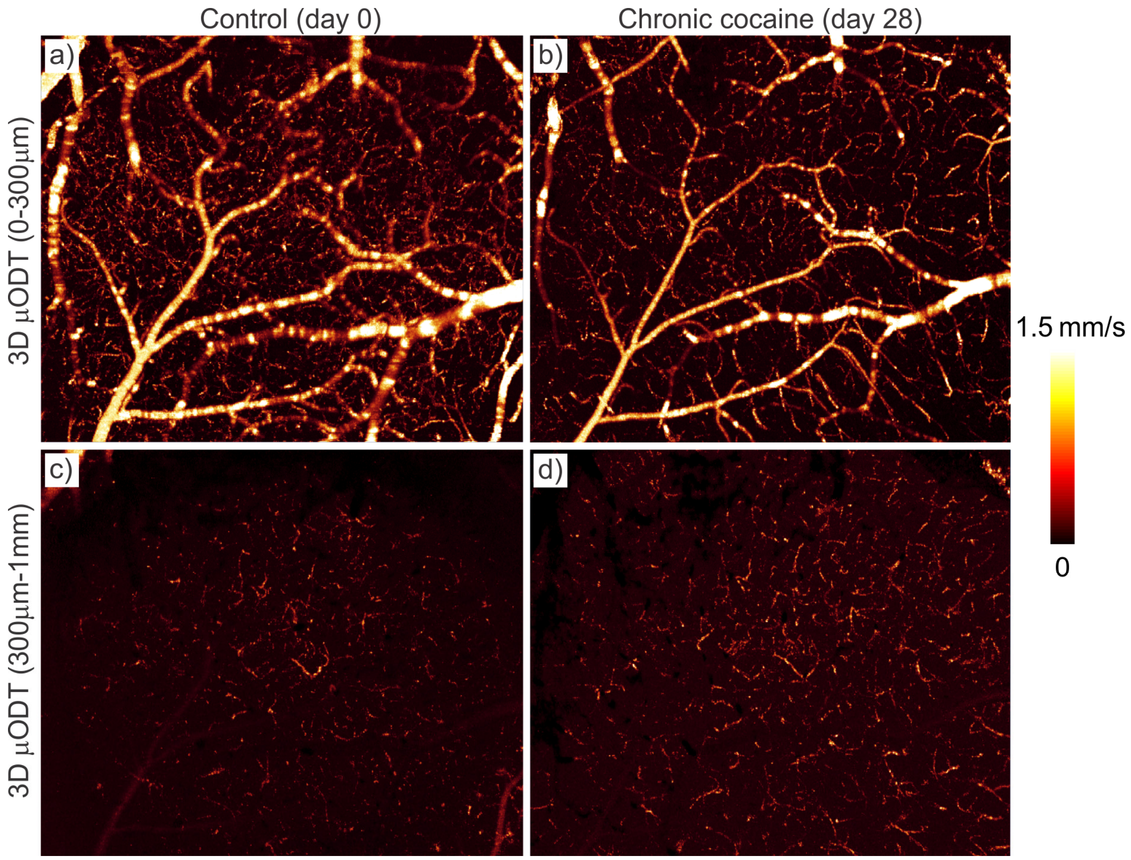

Supplementary Fig.s7 Chronic cocaine induced redistribution of the CBFv networks in the cortex imaged in an awake mouse. (**a**-**b**) 3D μODT images (2.3×2×0.3mm^3) in upper cortex (e.g., L1-L3) on day 0 (control) and day 28 (chronic cocaine), showing reduced capillary CBFv beds, reduced flows and vasoconstriction in AF and VF branches in chronic case (**b**); (**c**-**d**) the corresponding 3D μODT images (2.3×2×0.7mm^3) in deeper cortex (e.g., L3-L6 and below), showing increased CBFv in the capillary beds in the chronic case (**d**). Taking together, the results – similar to those in **Fig.9** - show chronic cocaine induced overall hypoperfusion and vascular redistribution in the cortical brain.
