# Supplementary material for: Dynamic 3D imaging of cerebral blood flow in awake mice using self-supervised-learning-enhanced optical coherence Doppler tomography
Source: Commun Biol. 2023 Mar 21;6:298. doi: 10.1038/s42003-023-04656-x (PMC10030663; doi:10.1038/s42003-023-04656-x)
Supplement: Supplementary file 3 — Description of Additional Supplementary Files [file 42003_2023_4656_MOESM3_ESM.pdf]

Description of Additional Supplementary Files

File name: Supplementary Video S1

Description: Animal movement before treadmill training in a head-restrained mouse.

File name: Supplementary Video S2

Description: Animal movement after treadmill training in a head-restrained mouse.

File name: Supplementary Data 1

Description: Datasets for plotting all the graphs in the article file

File name: Supplementary Data 2

Description: Datasets for plotting all the graphs in the supplementary information file
